# *α*-Lipoic Acid Strengthens the Antioxidant Barrier and Reduces Oxidative, Nitrosative, and Glycative Damage, as well as Inhibits Inflammation and Apoptosis in the Hypothalamus but Not in the Cerebral Cortex of Insulin-Resistant Rats

**DOI:** 10.1155/2022/7450514

**Published:** 2022-03-29

**Authors:** Mateusz Maciejczyk, Ewa Żebrowska, Miłosz Nesterowicz, Małgorzata Żendzian-Piotrowska, Anna Zalewska

**Affiliations:** ^1^Department of Hygiene, Epidemiology, And Ergonomics, Medical University of Bialystok, 2C Mickiewicza Street, 15-022 Bialystok, Poland; ^2^Department of Physiology, Medical University of Bialystok, 2C Mickiewicza Street, 15-022 Bialystok, Poland; ^3^Students Scientific Club “Biochemistry of Civilization Diseases” at the Department of Hygiene, Epidemiology and Ergonomics, Medical University of Bialystok, 2C Mickiewicza Street, 15-233 Bialystok, Poland; ^4^Department of Restorative Dentistry and Experimental Dentistry Laboratory, Medical University of Bialystok, 24A Marii Sklodowskiej-Curie Street, 15-276 Bialystok, Poland

## Abstract

The research determined the role of *α*-lipoic acid (ALA) in reducing the brain manifestations of insulin resistance. The mechanism of ALA action is mainly based on its ability to “scavenge” oxygen free radicals and stimulate biosynthesis of reduced glutathione (GSH), considered the most critical brain antioxidant. Although the protective effect of ALA is widely documented in various diseases, there are still no studies assessing the influence of ALA on brain metabolism in the context of insulin resistance and type 2 diabetes. The experiment was conducted on male Wistar rats fed a high-fat diet for ten weeks with intragastric administration of ALA for four weeks. We are the first to demonstrate that ALA improves the function of enzymatic and nonenzymatic brain antioxidant systems, but the protective effects of ALA were mainly observed in the hypothalamus of insulin-resistant rats. Indeed, ALA caused a significant increase in superoxide dismutase, catalase, peroxidase, and glutathione reductase activities, as well as GSH concentration and redox potential ([GSH]^2^/[GSSG]) in the hypothalamus of HFD-fed rats. A consequence of antioxidant barrier enhancement by ALA is the reduction of oxidation, glycation, and nitration of brain proteins, lipids, and DNA. The protective effects of ALA result from hypothalamic activation of the transcription factor Nrf2 and inhibition of NF-*κ*B. In the hypothalamus of insulin-resistant rats, we demonstrated reduced levels of oxidation (AOPP) and glycation (AGE) protein products, 4-hydroxynoneal, 8-isoprostanes, and 3-nitrotyrosine and, in the cerebral cortex, lower levels of 8-hydroxydeoxyguanosine and peroxynitrite. In addition, we demonstrated that ALA decreases levels of proinflammatory TNF-*α* but also increases the synthesis of anti-inflammatory IL-10 in the hypothalamus of insulin-resistant rats. ALA also prevents neuronal apoptosis, confirming its multidirectional effects within the brain. Interestingly, we have shown no correlation between brain and serum/plasma oxidative stress biomarkers, indicating the different nature of redox imbalance at the central and systemic levels. To summarize, ALA improves antioxidant balance and diminishes oxidative/glycative stress, protein nitrosative damage, inflammation, and apoptosis, mainly in the hypothalamus of insulin-resistant rats. Further studies are needed to determine the molecular mechanism of ALA action within the brain.

## 1. Introduction

In recent years, there has been a global epidemic of metabolic diseases such as obesity, insulin resistance, and type 2 diabetes. Approximately 8.8% (415 million) of adults aged 20-79 have type 2 diabetes, and by 2040, the number of patients will increase by more than 50% [1]. Overnutrition and lack of physical activity increase the incidence of not only metabolic and cardiovascular disorders. These include depression, cognitive dysfunction, dementia, Alzheimer's disease (AD), Parkinson's disease (PD), or amyotrophic lateral sclerosis (ALS). Although a diverse clinical picture characterizes both metabolic and neurodegenerative disorders, they are believed to be ruled by similar mechanisms of neurodegeneration [2–11]. The pathogenesis of type 2 diabetes involves two primary metabolic defects: impaired insulin secretion and insulin resistance, defined as a reduction of tissue insulin sensitivity caused by a loss/downregulation of the insulin receptors (IR) or insulin receptor substrates (IRS-1 and IRS-2) [12–14]. For many years, the brain was thought of as an “insulin-insensitive” organ; however, recent reports indicated that insulin could cross the blood-brain barrier (BBB) produced within the brain structures such as the hypothalamus and cerebral cortex [15–17]. A significant role in the etiology of metabolic and neurodegenerative disorders is also attributed to oxidative and nitrosative stress [18–20]. Induction of redox imbalance is related to the increase in glucose and free fatty acid oxidation, leading to the overproduction of reactive oxygen species (ROS) during cellular respiration in mitochondria. This process is also caused by nonenzymatic glycation of proteins—the resulting advanced glycation end products (AGE) increase ROS formation by inducing the NADPH oxidase activity [3, 18, 20–25]. NADPH oxidase is not only a primary source of free radicals but also an essential proinflammatory enzyme that enhances the secretion of many neuronal cytokines, including mainly tumor necrosis factor-alpha (TNF-*α*) [3, 10, 26, 27]. Despite several data on the association of oxidative stress and brain complications of type 2 diabetes [3, 18, 20], little is known about the disturbances in cerebral redox homeostasis. There is also a lack of studies comparing oxidative/nitrosative damage in various brain regions. Numerous experimental and clinical studies have become vital in explaining the brain's insulin resistance pathophysiology and developing new therapeutic strategies (preventing or eliminating these disorders) [18–20, 28–32].

Neurons are particularly vulnerable to oxidative damage. The brain consumes more oxygen than other organs, has less antioxidant enzyme activity, and contains more polyunsaturated fatty acids [33]. However, the brain is also a rich source of glutathione. This compound occurs in several forms, of which 99% is reduced glutathione (GSH). The remaining glutathione pool includes the oxidized glutathione (GSSG), S-nitrosoglutathione (GSNO), and glutathione disulfides [34]. As demonstrated in numerous studies, GSH plays a crucial role in maintaining the physiological redox balance of the brain. This compound is responsible for nonenzymatic and enzymatic inactivation of free radicals (as a component of glutathione peroxidase (GPx) and glutathione reductase (GR)) as well as participates in the regeneration of other antioxidants (e.g., vitamins C and E) and repair of oxidatively damaged proteins and lipids. GSH also maintains protein thiols in the reduced state and regulates gene expression (including inflammatory and insulin signaling pathways), as well as modulates neuronal differentiation and cell death. It is postulated that disturbances in glutathione metabolism may impair cerebral functioning in neurodegenerative diseases and diabetes [35–37]. Indeed, diminished glutathione concentration was observed in patients with AD, PD, ALS, and Huntington's disease [38–44]. Additionally, the results of recent studies indicated a significant decrease in GSH content in the insulin-resistant brain (with a simultaneous increase in GSSG concentration), which correlates with the degree of cerebral cognitive impairment [45–47]. It is suggested that GSH supplementation could improve brain functioning in prediabetes conditions [48]. Nevertheless, GSH does not pass through cell membranes and very poorly crosses the BBB [34]. Therefore, it is not surprising that new therapeutic strategies aimed at increasing the GSH level in the brain are still being sought.

One of the promising compounds that increase GSH production in the brain is *α*-lipoic acid (ALA) [49]. The results of experimental and clinical studies indicate the beneficial role of ALA in treating insulin resistance/type 2 diabetes at the central level. ALA supplementation has been observed to reduce plasma insulin concentration, improve peripheral sensitivity to this hormone, normalize glucose and triglyceride levels, and effectively prevent oxidative stress in insulin-resistant rats [50–52]. Despite the protective effect of ALA on lipid and carbohydrate metabolism, the mechanisms of ALA activity in the insulin-resistant brain are still not thoroughly explained. Bearing in mind the favorable properties of ALA in eliminating the peripheral effects of insulin resistance, we suppose that ALA may improve cerebral functions under prediabetes conditions. Recent studies indicate that ALA improves cognitive function in insulin-resistant rats. ALA normalizes insulin signaling, brain glucose metabolism, and synaptic plasticity in the cerebral cortex and hippocampus of diabetic rats [53]. However, little is known about the effects of ALA on brain redox homeostasis. In this paper, we are the first to evaluate the influence of ALA on cellular redox balance, oxidative and nitrosative stress, selected markers of inflammation and apoptosis, and their mutual interactions in the hypothalamus and cerebral cortex of insulin-resistant rats. We have examined the brain structures that regulate energy homeostasis and lipid/carbohydrate metabolism (hypothalamus), as well as the brain tissue responsible for the cognitive functions (cerebral cortex). In addition, our research is aimed at comparing disturbances in the redox balance at the systemic (plasma) and central (brain) levels.

## 2. Material and Methods

The investigation was approved by the Local Committee for Experiments on Animals in Olsztyn, Poland (approval no. 21/2017).

**Figure 1:**
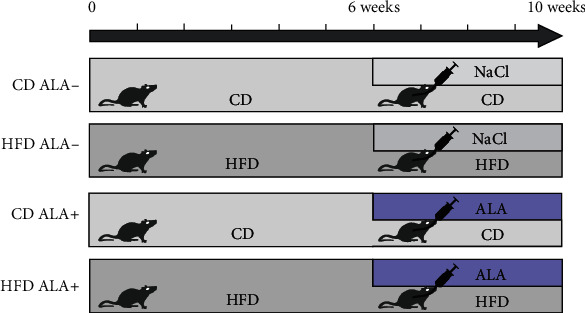
The outline of the experiment. CD ALA-: control animals not supplemented with ALA; HFD ALA-: high-fat diet-fed animals not supplemented with ALA; CD ALA+: control animals supplemented with ALA; HFD ALA+: high-fat diet-fed animals supplemented with ALA; NaCl: intragastric administration of saline; ALA: intragastric administration of *α*-lipoic acid.

The experiment was performed on 3-week-old male Wistar cmdb/outbred rats (with the initial body weight of approx. 50–70 g). The animals were kept in standard housing conditions for laboratory animals (21°C ± 2, 12 h light/12 h darkness) with unrestricted access to drinking water and food. After a week of adaptation to the environment, the animals were randomly divided into 4 groups of 10 individuals each (Figure 1):

1. Control (CD ALA-): for a 10-week period, the rats were fed with the control diet (CD; Agropol, Motycz, Poland) containing 10.3 kcal% fat, 24.2 kcal% protein, and 65.5 kcal% carbohydrate. After 6 weeks of the experiment, the animals additionally received intragastric saline solution for the next 4 weeks
2. HFD ALA-: for a period of 10 weeks, the rats were fed with the high-fat diet (D12492; Research Diets, Inc. New Brunswick, USA) containing 60 kcal% fat, 20 kcal% carbohydrate, and 20 kcal% proteins. After 6 weeks of the experiment, the animals additionally received intragastric saline solution for the next 4 weeks
3. CD ALA+: for a 10-week period, the rats were fed with the control diet. After 6 weeks of the experiment, the animals additionally received intragastric ALA solution at a dose of 30 mg/kg body weight for the next 4 weeks
4. HFD ALA+: for a period of 10 weeks, the rats were fed with the high-fat diet. After 6 weeks of the experiment, the animals additionally received intragastric ALA solution at a dose of 30 mg/kg body weight for the next 4 weeks

The ALA dose was chosen based on the literature data analysis [50–52]. 30 mg/kg body weight of ALA is one of the more commonly used ALA doses that do not cause toxic symptoms and have a good antioxidant effect. ALA was administered by intragastric route, which is the only way of administration guaranteeing that the animal takes a full dose of the drug. As demonstrated in our previous research, the intragastric route of administration is not associated with chronic stress to animals. The animals did not show any symptoms of pain or unusual behavior (mutilation, nervousness, compulsive behavior, changes in food and water intake, and changes in response to the external stimuli). The volume of 2 ml/kg body weight of the antioxidant solution and saline was administered every day at the same time. Food consumption and body weights were monitored every 3 days. Body mass index (BMI) was also analyzed using the weight and the height (the length from the tip of the nose to the anus). BMI was calculated using the formula BMI = body weight (g)/length^2^ (cm^2^) [54]. BMI between 0.45 g/cm^2^ and 0.65 g/cm^2^ are assumed to be normal values, whereas obesity is defined as BMI greater than 0.65 g/cm^2^. Lee index was determined using the formula Lee index = cube root of body weight (g)/length (cm) [54].

After 10 weeks, all animals were weighted and anesthetized with an intraperitoneal injection of phenobarbital (at a dose of 80 mg/kg body weight). The hypothalamus and cerebral cortex were taken by the same experienced technician, immediately after collection of blood from the abdominal aorta. Brain tissues were placed on ice and purified from blood elements and fat, then precooled in liquid nitrogen, and stored at -80°C until analysis. In order to assess insulin resistance, glucose and insulin concentrations were determined in the blood plasma (by ELISA method; EIAab, Wuhan, China). We also calculated the HOMA-IR index (homeostatic model assessment of *β*-cell function and insulin resistance) using the formula HOMA − IR index = fasting insulin (U/ml) × fasting glucose (mM)/22.5 [55].

Enzymatic antioxidants, glutathione metabolism, and oxidative modification products were evaluated in homogenates of the hypothalamus and cerebral cortex as well as in the plasma/serum samples. All the spectrophotometric and fluorimetric analyses were conducted using Infinite M200 PRO (Multimode Microplate Reader, Tecan) and standardized to one milligram of total protein.

### 2.1. Enzymatic Antioxidants

Activities of catalase (CAT, EC 1.11.1.6), glutathione peroxidase (GPx, EC 1.11.1.9), glutathione reductase (GR, E.C. 1.8.1.7), and superoxide dismutase-1 (SOD, EC 1.15.1.1) were analyzed.

Determination of CAT activity was based on the measurement of decomposition rate of hydrogen peroxide (H_2_O_2_) in 50 mM phosphate buffer at 340 nm according to method of Aebi [56]. One unit of CAT activity was defined as an amount of enzyme which degrades 1 *μ*mol of H_2_O_2_ per one minute. GPx activity was assessed by the method of Paglia and Valentine [57], measuring the conversion of NADPH to NADP^+^ at 340 nm. One unit of GPx activity was defined as the amount of enzyme catalysing oxidation of 1 mmol NADPH per one minute. GR activity was determined spectrophotometrically at 340 nm, with the use of Mize and Langdon's [58] method. One unit of enzyme activity was calculated as the amount of enzyme necessary for oxidation of 1 *μ*mol of NADPH per 1 minute. SOD activity was estimated according to the method of Misra and Fridovich [59]. One unit of SOD was defined as the amount of enzyme, which inhibits epinephrine oxidation to adrenochrome by 50%.

### 2.2. Glutathione Metabolism

Total glutathione, reduced glutathione (GSH), and oxidized glutathione (GSSG) as well as redox potential were investigated [60]. GSH was determined colorimetrically at 412 nm, basing on the reaction between NADPH, DTNB (5,5′-dithiobis-(2-nitrobenzoic acid)), and GR. For GSSG evaluation, prior to the analysis, the samples were thawed and neutralized to pH 6-7 with 1 M hydrochloric triethanolamine and subsequently incubated with 2-vinylpyridine. GSH level was calculated from the difference between the total glutathione and GSSG, whereas the redox status was evaluated using the formula [GSH]^2^/[GSSG] [61].

### 2.3. Oxidation and Glycation of Proteins

Content of protein carbonyl groups (PC) and advanced oxidation protein products (AOPP) as well as concentration of advanced glycation end products (AGE) was evaluated. The concentration of PC was measured by 2,4-dinitrophenylhydrazine (2,4-DNPH) according to the method of Reznick and Packer [62]. The principle of the method is based on the reaction of 2,4-DNPH with carbonyl groups in the oxidatively damaged proteins, which results in hydrazone, determined colorimetrically at 355 nm. PC content was calculated using an absorption coefficient for 2, 4‐DNPH = 22,000 M^−1^ cm^−1^. The content of AGE was determined fluorimetrically by measuring AGE-specific fluorescence at 350 nm excitation wavelength and 440 nm emission wavelength [63]. AOPP was measured based on changes in the absorbance caused by the iodine ion's oxidative capacity at 340 nm. For AGE and AOPP determination, plasma samples were diluted in phosphate-buffered saline (PBS, pH 7.2) 1 : 5 (*v* : *v*).

### 2.4. Oxidation of Lipids and DNA

4-Hydroxynonneal protein adducts (4-HNE), 8-isoprostanes, and 8-hydroxy-2′-deoxyguanosine (8-OHdG) concentrations were determined with an ELISA test using commercial kit protocols (Cell Biolabs, Inc., San Diego, CA, USA; Cayman Chemical, Ann Arbor, MI, USA; EIAab, Wuhan, China) in accordance with the manufacturer's instructions.

### 2.5. Inflammation and Apoptosis

Tumor necrosis factor-*α* (TNF-*α*) and interleukin 10 (IL-10) as well as caspase-3 (casp-3, EC 3.4.22.56) concentrations were assayed. TNF-*α* and IL-10 concentrations were assessed by ELISA, using commercial sets (EIAab, Wuhan, China) following the manufacturer's instructions. The activity of casp-3 was determined using Ac-Asp-Glu-Val-Asp-p-nitroanalide as a substrate [64]. The amount of p-nitroaniline (pNA) released by casp-3 activity was quantitated by measuring the absorbance at 405 nm.

### 2.6. Nitrosative Stress

Peroxynitrite (ONOO^−^) level was assayed according to the method described by Choromańska et al. [65]. The basis of the ONOO^−^ assay is peroxynitrite-mediated nitration of phenol resulting in nitrophenol formation. The concentration of 3-nitrotyrosine (3-NT) was assayed using the ELISA method, according to the manufacturer's protocol (Cell Biolabs, Inc., San Diego, CA, USA).

### 2.7. Real-Time PCR

Antioxidant response, inflammation, and neuronal apoptosis were also determined by real-time polymerase chain reaction (RT-PCR). For RNA purification, frozen brain samples were homogenized by grinding with liquid nitrogen and dissolved in isolation buffer provided in the NucleoSpin RNA/Protein kit (Macherey-Nagel, Düren, Germany). The RNA level and purity were checked at 260 nm and 280 nm. The synthesis of cDNA was performed using the EvoScript universal cDNA master kit (Roche Molecular Systems, Boston, MA, USA). The mRNA expression levels of *Nrf2* (qHsaCED0038543), *Nfκb* (qRnoCID0003698), *CAT* (qRnoCID0005259), *GR* (qRnoCID0007862), *GSS* (qRnoCED0005581), *TNF-α* (qRnoCED0009117), and *Casp-3* (qHsaCED0003013) were analyzed using the LightCycler® 96 Real-Time PCR System (Roche, Mannheim, Germany) with SYBR Green Supermix (Bio-Rad Laboratories, Hercules, CA), according to the manufacturer's instructions. GAPDH (qRnoCED0006459) was used as a housekeeping gene. The mRNA levels of target genes were normalized to GAPDH and calculated according to Pfaffl's method [66].

### 2.8. Statistical Analysis

GraphPad Prism 9 (GraphPad Software, La Jolla, CA, USA) for macOS was used to perform the statistical analysis. Homogeneity of variance was tested using Levene's test, while normality of distribution was checked using Shapiro-Wilk's test. For group comparisons, three-way analysis of variance ANOVA with Tukey adjustment was performed. Pearson's correlation coefficients were also used. The significance level was set at *p* ≤ 0.05. Values are given as mean ± standard deviation (± SD).

## 3. Results

### 3.1. General Animal Characteristics

Body weight, BMI, and Lee index of animals fed the HFD were significantly higher (+42%, +51%, and +208%, respectively) when compared to those of the control (Figure S1). ALA administration effectively reduced the abovementioned parameters to the level observed in the control group.

Fasting plasma glucose, insulin, and HOMA-IR were also elevated in HFD ALA- rats (+69%, +120%, and +271%, respectively) as compared to CD ALA- rats. ALA supplementation considerably decreased plasma glucose, insulin, and HOMA-IR in HFD animals when compared to HFD ALA- rats (-34%, -44%, and -63%), but their level was still higher than that observed in the CD ALA- group (+12%, +22%, and +37%) (Figure S1).

### 3.2. Brain Enzymatic Antioxidants

**Figure 2:**
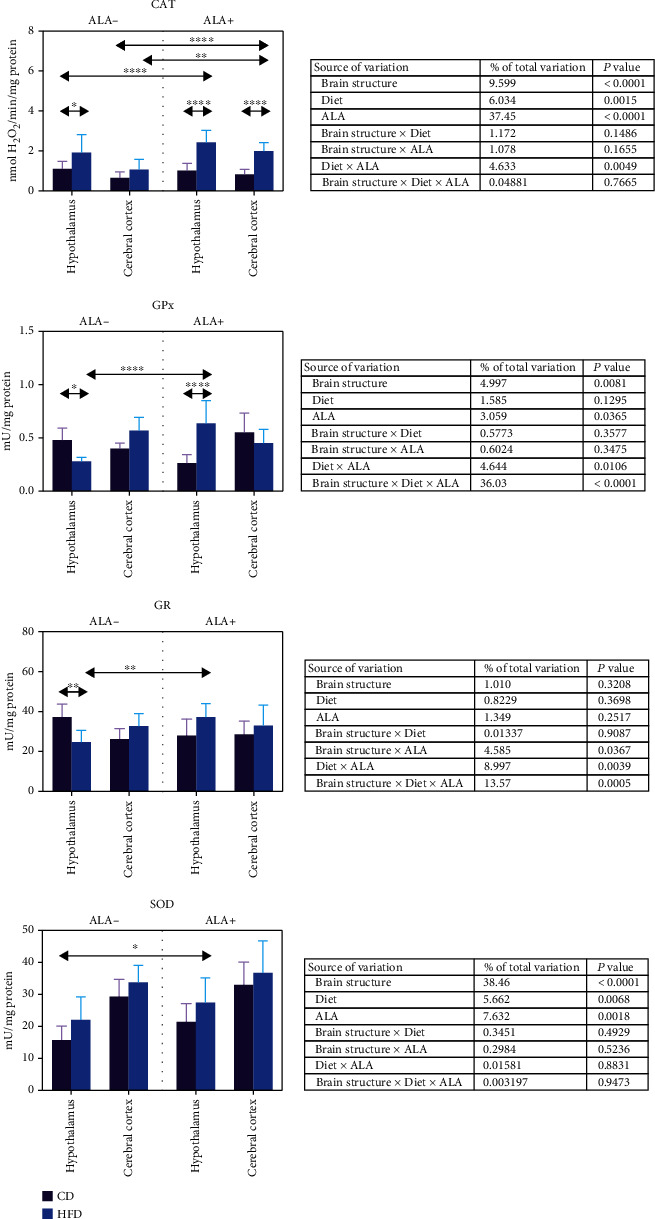
Effect of ALA supplementation on brain enzymatic antioxidant activity (CAT, GPx, GR, and SOD). Values are means ± SD, *n* = 10. Differences statistically significant at: ^∗^*p* < 0.05, ^∗∗^*p* < 0.005, ^∗∗∗^*p* < 0.0005, and ^∗∗∗∗^*p* < 0.0001. CAT: catalase; CD ALA-: control animals not supplemented with ALA; CD ALA+: control animals supplemented with ALA; GPx: glutathione peroxidase; GR: glutathione reductase; HFD ALA-: high-fat diet-fed animals not supplemented with ALA; HFD ALA+: high-fat diet-fed animals supplemented with ALA; SOD: superoxide dismutase-1.

The activity of enzymatic antioxidants in the cerebral cortex of ALA- animals was unaffected by HFD (Figure 2). CAT activity in the hypothalamus of HFD-fed animals was higher (+74%) when compared to that of control. In contrast, hypothalamic activity of GPx and GR was lower (–43% and –34%, respectively) in the HFD ALA- group in comparison to CD ALA-. ALA supplementation led to an increase in CAT activity both in the hypothalamus and cerebral cortex (+139%) of HFD animals when compared to CD. We have not observed any significant effect of ALA administration on CAT activity in HFD animals (when compared to HFD ALA-) in the hypothalamus whereas in the cerebral cortex, enzyme activity was higher (+86%) in the HFD group after ALA treatment. An increase of CAT activity after ALA supplementation was also observed in the HFD hypothalamus and cerebral cortex (+120% and +206%, respectively) when compared to CD ALA- animals. After ALA supplementation, an increase in GPx activity was also observed but only in the hypothalamus of HFD animals when compared to CD ALA+ (+142%) and HFD ALA- (+127%). Hypothalamic GR activity in HFD animals treated with ALA was also increased (+51%) but only when compared to the HFD ALA- group. SOD activity was not affected by HFD regime. We have only observed an increase in SOD activity (+75%) in the hypothalamus of HFD ALA+ when compared to CD ALA- (Figure 2).

Three-way ANOVA indicated that CAT and SOD activity was mostly influenced by ALA supplementation, the studied brain structure as well as the diet (alone and for CAT—together with ALA) (Figure 2). GR in contrast was affected mainly by combination of the brain structure, diet, and ALA treatment and diet and ALA treatment as well as brain structure and ALA (but not the diet) interaction.

### 3.3. Brain Glutathione Metabolism

**Figure 3:**
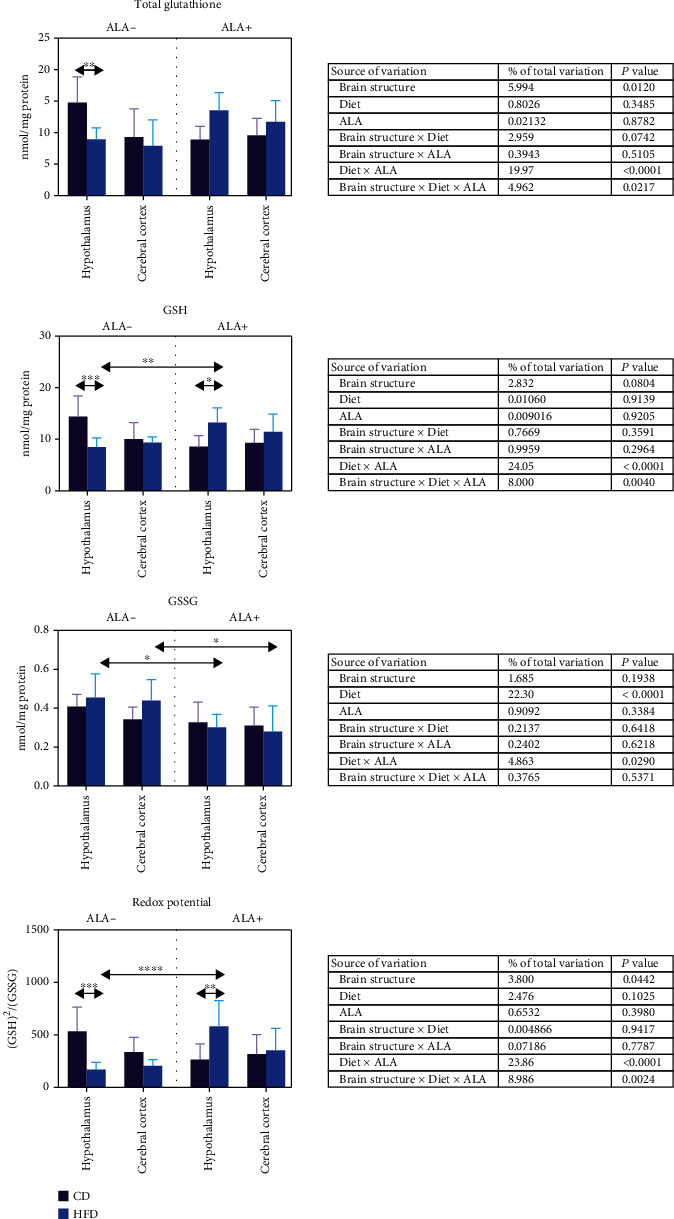
Effect of ALA supplementation on brain glutathione metabolism (total glutathione, GSH, GSSG, and redox potential). Values are means ± SD, *n* = 10. Differences statistically significant at ^∗^*p* < 0.05, ^∗∗^*p* < 0.005, ^∗∗∗^*p* < 0.0005, and ^∗∗∗∗^*p* < 0.0001. CD ALA-: control animals not supplemented with ALA; CD ALA+: control animals supplemented with ALA; GSH: reduced glutathione; GSSG: oxidized glutathione; HFD ALA-: high-fat diet-fed animals not supplemented with ALA; HFD ALA+: high-fat diet-fed animals supplemented with ALA.

In HFD animals, we have observed a lowered level of total glutathione and GSH as well as the redox potential (-39%, -41%, and -68%, respectively) but only in the hypothalamus when compared to CD ALA- (Figure 3). We have not observed any effect of ALA supplementation on total glutathione concentration. In contrast, ALA-treated HFD animals expressed a higher GSH content and redox potential in the hypothalamus in comparison to the HFD ALA- group (+56% and +242%, respectively) and in comparison to the CD ALA+ group (+54% and +120%).

GSSG content remained almost unchanged during the study—except its lowered content after ALA administration in HFD animals both in the hypothalamus and cerebral cortex when compared to HFD ALA- animals (-34% and -37%, respectively) (Figure 3).

Three-way ANOVA showed that the diet in combination with ALA administration expressed the strongest effect on glutathione metabolism (Figure 3).

### 3.4. Brain Oxidation and Glycation of Proteins

**Figure 4:**
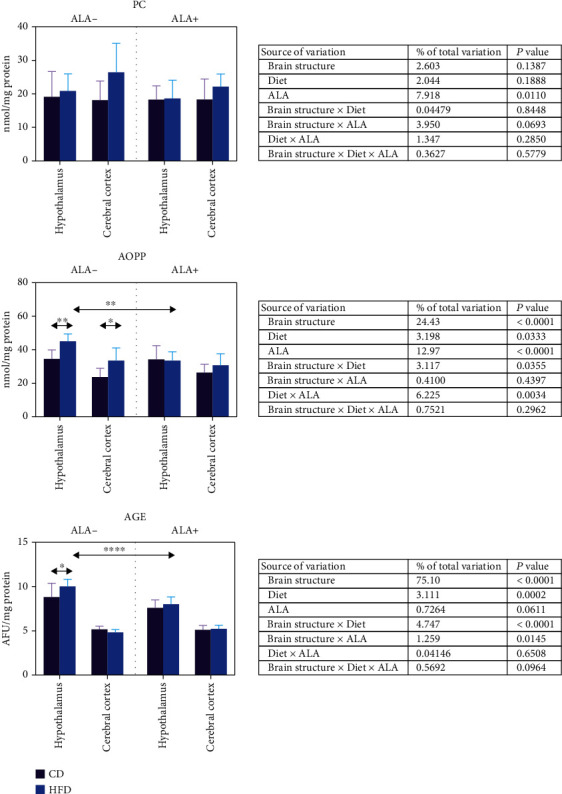
Effect of ALA supplementation on brain oxidation and glycation of proteins (PC, AOPP, and AGE). Values are means ± SD, *n* = 10. Differences statistically significant at ^∗^*p* < 0.05, ^∗∗^*p* < 0.005, and ^∗∗∗∗^*p* < 0.0001. AGE: advanced glycation end products; AOPP: advanced oxidation protein products; CD ALA-: control animals not supplemented with ALA; CD ALA+: control animals supplemented with ALA; HFD ALA-: high-fat diet-fed animals not supplemented with ALA; HFD ALA+: high-fat diet-fed animals supplemented with ALA; PC: protein carbonyl groups.

We have not observed any differences in PC concentration between all the studied groups (Figure 4). AOPP content in the hypothalamus and cerebral cortex of HFD ALA- animals was higher (+31% and +41%, respectively) when compared to control. AGE level was also higher in HFD animals, but only in the hypothalamus (+14%). Interestingly, ALA supplementation lowered AOPP (-26%) and AGE (-20%) concentration in in the hypothalamus of HFD animals in comparison to HFD ALA animals (Figure 4).

Three-way ANOVA indicated that AOPP content was mostly influenced by brain structure, then ALA supplementation, diet and ALA, diet (solely), and brain structure in combination with diet (Figure 4). Similarly, AGE concentration was mostly dependent of the brain structure, then brain structure and diet and diet and brain structure combined with ALA supplementation.

### 3.5. Brain Oxidation of Lipids and DNA

**Figure 5:**
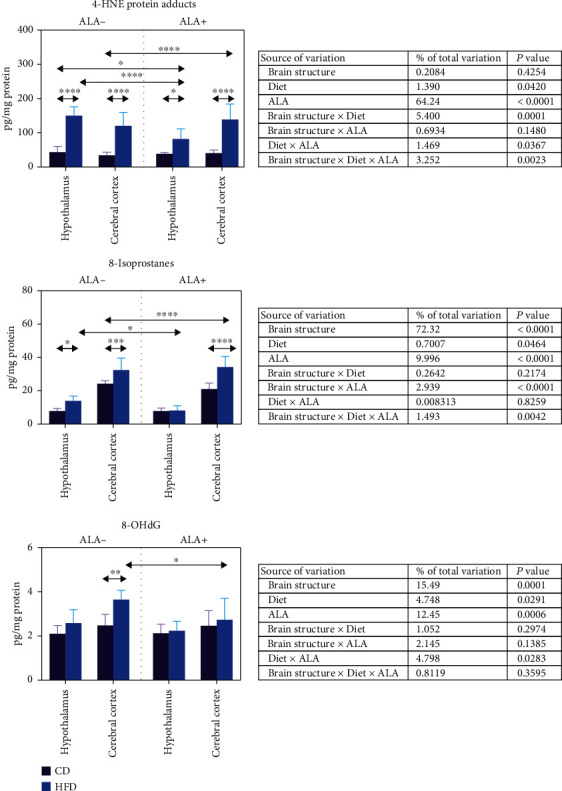
Effect of ALA supplementation on brain oxidation of lipids (4-HNE and 8-izoprostanes) and DNA (8-OHdG). Values are means ± SD, *n* = 10. Differences statistically significant at ^∗^*p* < 0.05, ^∗∗∗^*p* < 0.0005, and ^∗∗∗∗^*p* < 0.0001. CD ALA-: control animals not supplemented with ALA; CD ALA+: control animals supplemented with ALA; HFD ALA-: high-fat diet-fed animals not supplemented with ALA; HFD ALA+: high-fat diet-fed animals supplemented with ALA; 4-HNE: 4-hydroxynonneal protein adducts; 8-OHdG: 8-hydroxy-2′-deoxyguanosine.

4-HNE protein adduct and 8-isoprostane content was higher in the hypothalamus (+244% and +77%, respectively) and cerebral cortex (+251% and +34%) of HFD ALA- animals when compared to control (Figure 5). 8-OHdG was higher only in the cerebral cortex of the ALA- HFD group in comparison to CD. In the hypothalamus of HFD animals, ALA supplementation led to an increase in 4-HNE concentration when compared to CD ALA+ (+113%) and CD ALA- (+88%) and a decrease (-44%) when compared to HFD ALA-. In the cerebral cortex, we have not observed any effect of ALA supplementation in the HFD group; however, we noticed higher 4-HNE content when compared to CD ALA+ (+246%) and CD ALA- (+304%) animals. ALA administration to HFD animals led to a decrease in the hypothalamic 8-isoprostanes (-42%) as compared to the HFD ALA- group and an increase in the cerebral cortex 8-isoprostanes when compared to CD ALA+ (+62%) and to the CD ALA- group. ALA supplementation to HFD animals diminished 8-OHdG concentration only in the cerebral cortex (-25%) when compared to HFD ALA- (Figure 5).

According to three-way ANOVA, 4-HNE content was mainly influenced by ALA supplementation, brain structure and diet, brain structure in combination with diet and ALA, and diet and ALA as well as diet. In contrast, 8-isoprostanes, an 8-OHdG content, was mostly dependent on brain structure and ALA (Figure 5).

### 3.6. Brain Inflammation and Apoptosis

**Figure 6:**
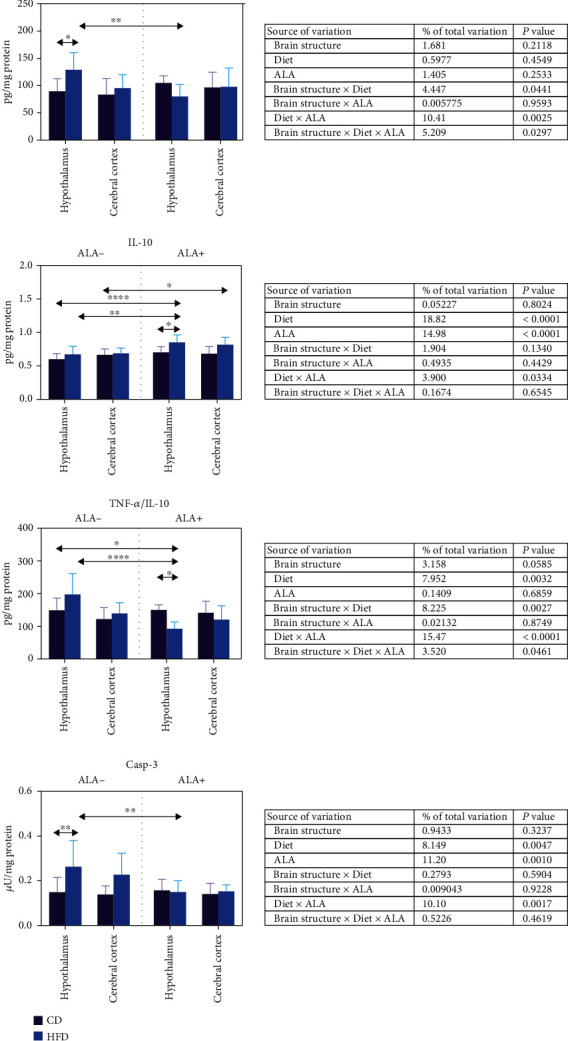
Effect of ALA supplementation on brain inflammation and apoptosis (TNF-*α*, IL-10, TNF-*α*/IL-10 ratio, and casp-3). Values are means ± SD, *n* = 10. Differences statistically significant at ^∗^*p* < 0.05, ^∗∗^*p* < 0.005, and ^∗∗∗∗^*p* < 0.0001. casp-3: caspase-3; CD ALA-: control animals not supplemented with ALA; CD ALA+: control animals supplemented with ALA; HFD ALA-: high-fat diet-fed animals not supplemented with ALA; HFD ALA+: high-fat diet-fed animals supplemented with ALA; IL-10: interleukin 10; TNF-*α*: tumor necrosis factor-*α*.

In the hypothalamus of HFD animals TNF-*α* and casp-3 content was increased (+44% and+77%, respectively); however, ALA supplementation significantly decreased their concentrations (-38% and -43%) when compared to HFD ALA- animals (Figure 6). On the other hand, we have not observed any changes in IL-10 content in the HFA ALA animals, not supplemented with ALA. However, in the hypothalamus of HFD ALA+ rats, IL-10 concentration was higher than in CD ALA+ (+22%), HFD ALA- (+27%), and CD ALA- (+42%). In the cerebral cortex of HFD ALA+ animals, we only observed an increased IL-10 content (+23%) when compared to CD ALA- rats. ALA treatment alleviated TNF-*α*/IL-10 ratio in the hypothalamus of HFD animals when compared to CD ALA+ (-39%), HFD ALA- (-53%), and CD ALA- (-38%) (Figure 6).

Three-way ANOVA indicated that IL-10 and casp-3 content was mostly influenced by the diet and ALA supplementation (solely and in combination with each other). TNF-*α*/IL-10 ratio was mostly dependent of the diet in combination with ALA, then brain structure and diet, as well as solely by the diet (Figure 6).

### 3.7. Brain Nitrosative Stress

**Figure 7:**
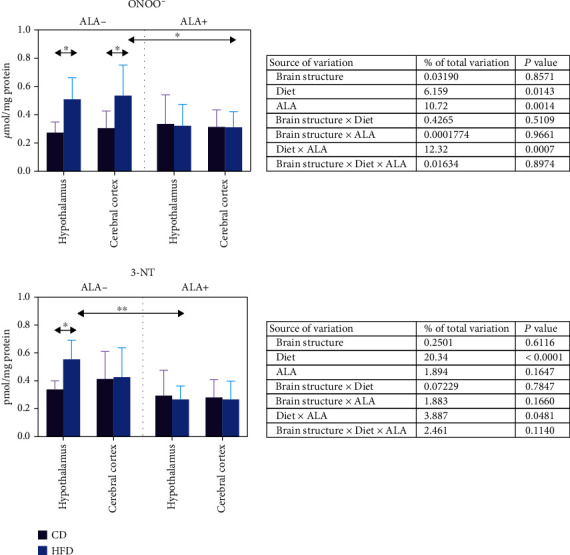
Effect of ALA supplementation on brain nitrosative stress (ONOO^−^ and 3-NT). Values are means ± SD, *n* = 10. Differences statistically significant at ^∗^*p* < 0.05 and ^∗∗^*p* < 0.005. ALA-: control animals not supplemented with ALA; CD ALA+: control animals supplemented with ALA; HFD ALA-: high-fat diet-fed animals not supplemented with ALA; HFD ALA+: high-fat diet-fed animals supplemented with ALA; ONOO^−^: peroxynitrite; 3-NT: 3-nitrotyrosine.

ONOO^−^ content was markedly higher in the hypothalamus (+86%) and cerebral cortex (+75%) of HFD ALA- animals when compared to control (Figure 7). ALA supplementation alleviated these changes, but only in the cerebral cortex (-42% when compared to HFD ALA- animals). In contrast, we observed an increased 3-NT content only in the hypothalamus of HFD animals (+86%) when compared to CD. ALA treatment alleviated 3-NT content in the hypothalamus of HFD animals when compared to HFD ALA- (-52%) (Figure 7).

Three-way ANOVA indicated that 3-NT content was mostly influenced by the diet alone and when combined with ALA whereas ONOO^−^ concentration was affected by the diet and ALA treatment and solely the diet or ALA supplementation (Figure 7).

### 3.8. Real-Time PCR

**Figure 8:**
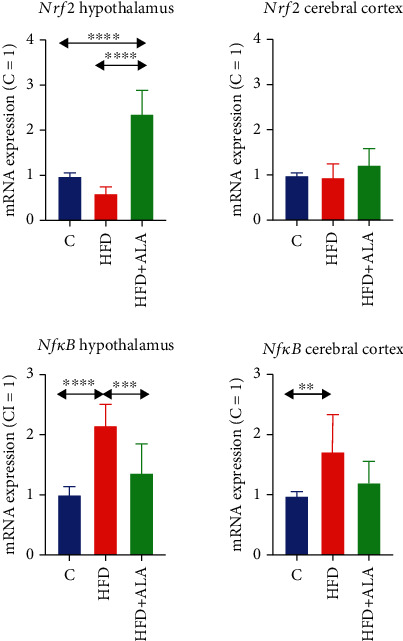
Effect of ALA supplementation on brain transcription factor expression (*Nrf2* and *Nfκb*). Values are means ± SD, *n* = 8. Differences statistically significant at ^∗∗^*p* < 0.005, ^∗∗∗^*p* < 0.0005, and ^∗∗∗∗^*p* < 0.0001. C: control animals not supplemented with ALA; HFD: high-fat diet-fed animals not supplemented with ALA; HFD+ALA: high-fat diet-fed animals supplemented with ALA; *Nfκb*: nuclear factor kappa-light-chain-enhancer of activated B cells; Nrf2: nuclear factor- (erythroid-derived 2-) like 2.

*Nrf2* mRNA expression was markedly higher in the hypothalamus after ALA treatment, when compared to control (+136%) and HFD (+295%), whereas in the cerebral cortex, its level remained unchanged in all the studied groups (Figure 8). The expression of *Nfκb* was higher in the hypothalamus of HFD animals when compared to the control (+115%); however, ALA treatment reduced its level (-37% vs. HFD) to that observed in the control. In the cerebral cortex of HFD animals, *Nfκb* expression was increased when compared to the control (+72%) (Figure 8).

**Figure 9:**
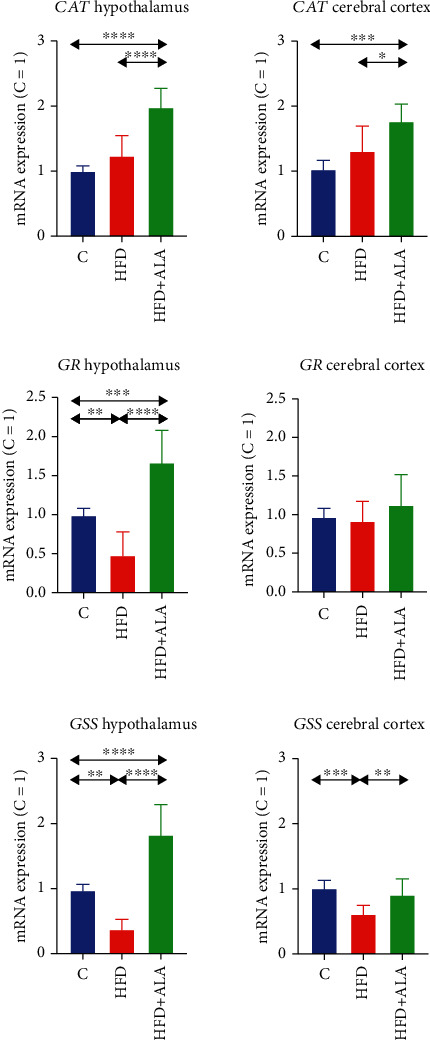
Effect of ALA supplementation on brain mRNA expression of antioxidant enzymes (*CAT*, *GSR*, and *GSS*). Values are means ± SD, *n* = 8. Differences statistically significant at ^∗∗^*p* < 0.005, ^∗∗∗^*p* < 0.0005, and ^∗∗∗∗^*p* < 0.0001. C: control animals not supplemented with ALA; CAT: catalase; *GSR*: glutathione reductase; *GSS*: glutathione synthase; HFD: high-fat diet-fed animals not supplemented with ALA; HFD+ALA: high-fat diet-fed animals supplemented with ALA.

The expression of *CAT* was significantly increased after ALA supplementation both in the hypothalamus and the cerebral cortex when compared to the control (+98% and +77%, respectively) and to the HFD animals (+60% and +35%) (Figure 9).

In the hypothalamus of HFD animals, the mRNA expression of *GSR* and *GSS* was lower when compared to the control (-52% and -63%, respectively). However, after ALA treatment, the level of the abovementioned genes markedly increased when compared to the control (+67% and 83%, respectively, for *GSR* and *GSS*) and when compared to HFD animals (+248% and +390%). In the cerebral cortex, the *GSS* expression was lower in the HFD group when compared to the control (-39%). ALA treatment led to an increase in the level of *GSS* in comparison to HFD rats (+49) (Figure 9).

**Figure 10:**
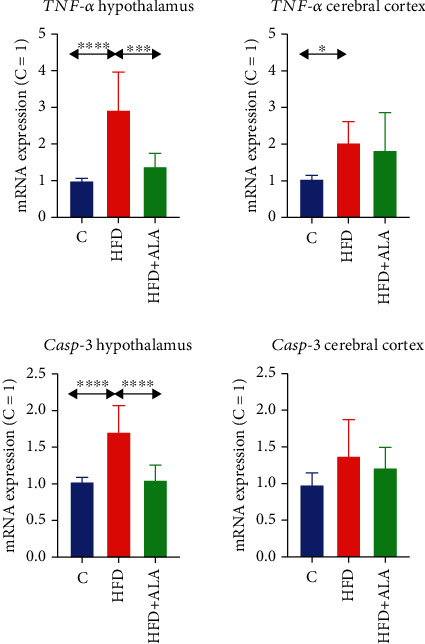
Effect of ALA supplementation on brain mRNA expression of cytokines (*TNF-α*) and apoptotic proteins (*Casp-3*). Values are means ± SD, *n* = 8. Differences statistically significant at ^∗^*p* < 0.05, ^∗∗∗^*p* < 0.0005, and ^∗∗∗∗^*p* < 0.0001. C: control animals not supplemented with ALA; *Casp-3*: caspase 3; HFD: high-fat diet-fed animals not supplemented with ALA; HFD+ALA: high-fat diet-fed animals supplemented with ALA; *TNF*-*α*: tumor necrosis factor-alpha.

*TNF-α* and *casp-3* expression was markedly higher in the hypothalamus of HFD animals when compared to the control (+193% and 71%, respectively); however, ALA supplementation reduced their level (-53% and -38%, vs. HFD) to that observed in the control. In the cerebral cortex of HFD animals, *TNF-α* expression was higher only in the HFD animals when compared to the control (Figure 10).

### 3.9. Plasma/Serum Enzymatic and Nonenzymatic Antioxidants

The activity of CAT, GR, and SOD was significantly lowered in the serum of HFD ALA- animals (-53%, -27%, and -61%, respectively) when compared to control (Figure S3). Supplementation of ALA to the HFD group markedly increased the plasma activity of CAT and SOD when compared to HFD ALA- animals (+66% and +98%, respectively) but to the level still lower than that observed in CD ALA- animals (-23%). ALA administration to HFD animals restored the activity of serum GR to the level observed in the CD ALA- rats (+36% vs. HFD ALA-). In contrast, GPx activity was not affected by HFD but ALA supplementation led to increased GPx activity in HFD ALA+ rats when compared to CD ALA- (+28%) and HFD ALA- (+26%) (Figure S2).

HFD significantly decreased the level of plasma GSH and redox potential (-49% and -78%, respectively) when compared to control. ALA administration efficiently alleviated negative outcomes of HFD and increased the level of GSH and redox potential to the level observed in CD ALA- animals (+107% and +430%, respectively) (Figure S2).

### 3.10. Plasma Oxidative Damage Markers

HFD led to an increase in plasma level of all the assayed oxidative damage markers: PC, AOPP, AGE, 4-HNE, 8-isoprostanes, and 8-OHdG (+152%, +62%, +92%, +140%, +117%, and +127%, respectively) as compared to control animals (Figure S2). ALA administration efficiently alleviated negative outcomes of HFD and reduced the level of PC, AGE, and 8-OHdG to the level observed in CD ALA- animals (-46%, -34%, and -41%, respectively). Supplementation of ALA to the HFD group also markedly decreased the plasma content of 4-HNE when compared to HFD ALA- animals (-15%) but to the level still higher than that observed in CD ALA- animals (+103%). We have not observed any positive effect of ALA treatment on plasma AOPP and 8-isoprostane level (Figure S3).

### 3.11. Correlations

We did not show any statistically significant correlations between brain and blood redox biomarkers.

## 4. Discussion

Our previous studies showed that both HFD and high-sucrose diet (HSD) enhance neuronal NADPH oxidase and disrupt the brain antioxidant systems, leading to intensified oxidation of cerebral proteins, lipids, and nucleic acids [18, 20]. Of particular note is the increase in hypothalamic uric acid (UA), which has a robust prooxidant effect at a high concentration. UA can generate free radicals (e.g., aminocarboline radical) or alkylated derivatives (e.g., in reaction with peroxynitrite) that irreversibly damage neuronal biomolecules. We have shown that both brain-level abnormalities and systemic disturbances in carbohydrate and lipid metabolism can be the source of increased oxidation in the hypothalamus and cerebral cortex [18, 20]. Indeed, under hyperglycemic conditions, glucose metabolism via the polyol pathway is increased, leading to a decrease in the NADPH/NADP^+^ ratio, thereby reducing the intracellular NADPH required for GSH biosynthesis [67, 68]. Induction of the polyol pathway and nonenzymatic glycation also lead to increased diacylglycerol (DAG) synthesis, resulting in the activation of protein kinase C and promoting arachidonic acid metabolism and nitric oxide (NO) release [69, 70]. The present study confirms the previous observations. In the hypothalamus of insulin-resistant rats, we showed a decrease in GSH, redox potential, and GR activity accompanied by increased oxidative (↑AOPP, ↑4-HNE, and ↑8-isoprostanes) and glycative (↑AGE) damage, as well as enhanced inflammation (↑TNF-*α*) and neuronal apoptosis (↑casp-3). Although we did not directly assess the rate of free radical production, it may be evidenced by an increase in CAT activity accompanied by a decrease in GPx activity. Although both CAT and GPx are involved in the degradation of hydrogen peroxide (H_2_O_2_), at high concentrations, this role is played by CAT (Michaelis–Menten constant (Km) for GPx = 1 × 10^−6^ M and CAT = 2.4 × 10^−4^ M) [71, 72]. Thus, excessive concentrations of hypothalamic H_2_O_2_ may cause oxidation of the active center of GPx and thus a decrease in enzyme activity [73]. In the brain, particularly large amounts of hydrogen peroxide are produced by monoamine oxidase (MAO) via oxidative deamination of monoamines (e.g., dopamine) [74]. Under GSH deficiency and high levels of transition metal ions (e.g., Fe^2+^ and Cu^2+^), H_2_O_2_ cannot be removed and, in the Fenton reaction, can serve as a substrate for the formation of hydroxyl radical (^·^OH). ^·^OH is particularly dangerous to neurons and glial cells since it has the strongest oxidation potential of all radicals [75–77]. Interestingly, in the cerebral cortex of insulin-resistant rats, we did not observe any changes in the efficiency of antioxidant systems, resulting in increased oxidative damage to proteins (↑AOPP), lipids (↑4-HNE, ↑8-isoprostanes), and DNA (↑8-OHdG). This confirms our previous reports of weaker antioxidant defense of the cerebral cortex [18–20]. Although this topic requires further research and observations, it may be due to the increased oxidative activity of cortical mitochondria or the higher content of transition metal ions than the hypothalamus [78, 79].

Decreased GSH level in the hypothalamus of insulin-resistant rats is most clinically relevant. Although the brain GSH system may be activated as an adaptive response to oxidative stress, with disease duration and continued ROS overproduction, antioxidant reserves may be depleted (↓GPx, ↓GR), and neuronal metabolism may be impaired [45–47]. A decrease in GSH concentration has been shown to activate brain lipoxygenase 12, leading to hydrogen peroxide formation, Ca^2+^ influx into the cell, and ultimately neuronal apoptosis (↑casp-3) [80–82]. Reduced GSH concentrations are observed in many systemic diseases. The best-documented observations concern neurodegenerative diseases (e.g., AD, PD, ALS, dementia, and cognitive impairment), diabetes, and its metabolic complications [34, 42, 43, 83–85]. Lowered GSH levels also correspond to cognitive dysfunction [45–47]. Recent studies indicate that GSH supplementation could improve brain functioning under insulin resistance conditions [48, 86–89]. Nevertheless, GSH does not pass through cell membranes and very poorly crosses the BBB. After oral administration, GSH is rapidly digested in the gastrointestinal tract, which prevents its use in clinical practice [34]. It is not surprising that new therapeutic strategies to increase the brain GSH are still being sought. This problem is the subject of interdisciplinary studies in medical biology, biochemistry, pharmacology, and clinical medicine and is attempted to be solved by supplementation with natural/synthetic glutathione derivatives as well as stimulation of GSH biosynthesis. Although the primary factor limiting the GSH production is the availability of cysteine, this amino acid—due to its high neurotoxicity—cannot be used in clinical practice [90]. One of the promising compounds that increase GSH biosynthesis is *α*-lipoic acid (ALA).

ALA, chemically named 1,2-dithiolane-3-pentanoic acid, is an 8-carbon, cyclic disulfide antioxidant with the formula C_8_H1_4_O_2_S_2_ and molecular weight 206.33 g/mol. Due to a nonpolar aliphatic chain and a carboxyl group, ALA has both hydrophobic and hydrophilic properties. This amphipathic character is unique among the antioxidants [49, 91]. Therefore, ALA readily crosses the cellular membranes and the BBB. After oral administration, ALA is rapidly and almost completely absorbed from the gastrointestinal tract. The maximum concentration in plasma is assessed 30 min after administration [50, 51, 92–94]. In living organisms, ALA is reduced to its dithiol form called dihydrolipoic acid (DHLA). ALA and DHLA create a potent redox couple, with a redox potential of –0.32 V. Considering that the redox potential of the GSH/GSSG system is –0.24 V, ALA/DHLA guarantees more effective protection against ROS than reduced glutathione! Therefore, the ALA/DHLA system is considered the “universal antioxidant” [50, 51, 92–94]. Although the protective effect of ALA is widely documented in various diseases, there are still no studies assessing the influence of ALA on brain metabolism in patients with insulin resistance and type 2 diabetes.

We are the first to show that ALA improves brain enzymatic and nonenzymatic antioxidant systems; however, we observed protective effects of ALA mainly in the hypothalamus of insulin-resistant rats. Indeed, ALA caused a significant increase in CAT, GPx, GR, and SOD activity, but also in GSH concentration and redox potential ([GSH]^2^/[GSSG]) in the hypothalamus of HFD-fed rats. ALA also reduces the hypothalamic GSSG level, while in the cerebral cortex, it only enhances CAT activity and decreases GSSG content. Interestingly, ALA can act both directly and indirectly. ALA reacts with radical and nonradical ROS by converting to the radical cation ALA^+·^ (e.g., ALA +  ^·^OH⟶ALA^+·^ + OH^−^), which is much less reactive than other radicals [91]. The resulting cation is readily converted back by other ROS scavengers (e.g., vitamins C and E), which in turn can be regenerated by DHLA. These processes occur in both membranes and the aqueous phase, which is particularly important for cerebral tissue. ALA also enhances the effects of other brain antioxidants (e.g., glutathione and coenzyme Q10) by reducing their radical or oxidized forms (e.g., DHLA + GSSG⟶ALA + 2GSH) [95–97]. ALA can also induce the expression of nuclear factor- (erythroid-derived 2-) like 2 (Nrf2) in peripheral tissues [98]. In our study, we are the first to show that ALA increases *Nrf2* expression in the hypothalamus of insulin-resistant rats. Nrf2 regulates the expression of many genes involved in antioxidant, anti-inflammatory, and cytoprotective processes, as well as regulating cell metabolism and mitochondrial bioenergetics [99, 100]. Since Nrf2 directly stimulates genes responsible for glutathione biosynthesis (↑*GSS*) [99, 101], it is not surprising to find an increase in the hypothalamic GSH with a concomitant decrease in the GSSG level [45–47]. Increasing the glutathione pool may be the key to improving brain metabolism under insulin resistance and diabetes [48, 86–89]. As the major brain antioxidant, GSH reacts directly with free radicals like superoxide radical (O_2_^-·^), ^·^OH, and ^·^NO. In the reaction catalyzed by GPx, GSH is also an electron donor for O_2_^-·^ reduction, thereby removing H_2_O_2_ and organic hydroperoxides. The resulting GSSG is then reduced by GR, which restores the brain GSH pool [34]. Although GSH is not consumed in reactions catalyzed by GPx and GR, glutathione regeneration requires a constant supply of NADPH. The primary intracellular source of NADPH is the pentose phosphate cycle [67]. Although it is impaired under hyperglycemia and insulin resistance, recent findings indicate that ALA increases NADPH availability, which may explain the higher GSH biosynthesis in the brain of insulin-resistant rats [102–104]. It should be noted that glutathione exhibits not only antioxidant activity. It also regulates gene expression (including inflammatory and insulin signaling pathways) and maintains thiol groups in the reduced state, as well as acts as a neurotransmitter, neuromodulator, or regulator of neuronal apoptosis [34]. However, ALA supplementation may also improve cognitive function under insulin resistance conditions. Indeed, we showed a significant decrease in GSSG level in the cerebral cortex of HFD-fed rats. In the neurodegenerative brain, there is nonspecific binding of transition metal ions by misfolded proteins (A*β* peptide in AD, *α*-synuclein in PD, and copper-zinc-superoxide dismutase (CuZnSOD) in ALS) [105, 106]. The rate-limiting step of protein folding is the oxidation of thiol residues of cysteine with the formation of -SS- bridges stabilizing the spatial structure of the protein. The primary oxidant of -SH groups is GSSG; thus, its decrease in the cerebral cortex may improve the cognitive function of rats [107, 108]. It should be noted that ALA and DHLA form stable complexes with transition metal ions, thus decreasing the intensity of oxidative protein modifications. Metal ions can also enter into oxidation-reduction reactions that, in an uncontrolled way, lead to the formation of the highly reactive ^·^OH. Therefore, chelation of metal ions by ALA may attenuate neuronal oxidative stress [109–111]. Unfortunately, due to lack of ethics committee approval, we did not conduct behavioral studies assessing cerebral cognition of rats. Further research is required; however, as we show here, ALA improves the glutathione system in both analyzed brain structures of HFD-fed rats. Recent studies also confirm that ALA improves cognitive function by normalizing brain glucose metabolism and the expression of synaptic plasticity proteins [53, 112].

**Figure 11:**
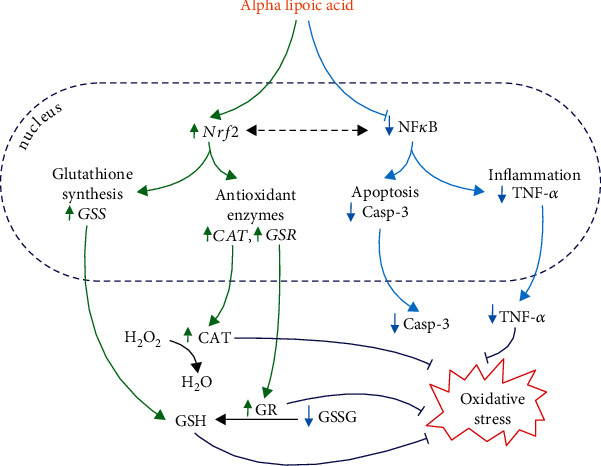
Postulated mechanism of ALA supplementation on hypothalamic metabolism in insulin-resistant rats.

Nrf2 activates the transcription of many cytoprotective genes [99, 100], which may explain the increased mRNA expression of *GSR* and *CAT*, as well as the higher activity of their protein products (↑GR, ↑CAT) in the hypothalamus of insulin-resistant rats (Figure 11). The consequence of strengthening the antioxidant barrier (↑CAT, ↑GPx, ↑GR, ↑SOD, ↑GSH, ↑redox potential, and ↓GSSG) by ALA is reduced oxidation, glycation, and nitration of brain proteins, lipids, and DNA [91, 113]. Thus, it is not surprising that we observed particularly beneficial effects in the hypothalamus of insulin-resistant rats (↓AOPP, ↓AGE, ↓4-HNE, ↓8-isoprostanes, and ↓3-NT) compared to the cerebral cortex (↓8-OHdG, ↓ONOO^−^). However, the favorable actions of ALA may also result from other mechanisms. Neuronal studies have shown that ALA enhances the activity of glutathione S-transferase (GST), thus degrading the lipid peroxides formed in the lipid peroxidation processes [114, 115]. An increase in GSH concentration and [GSH]^2^/[GSSG] ratio by ALA also enhances regeneration of vitamins C and E. DHLA can directly upregulate ascorbate, participating in its reconstitution from dehydroascorbate, as well as regenerate ubiquinol [91, 113]. This increases the antioxidant potential of the brain and prevents oxidative modifications of neuronal biomolecules [116]. However, oxidative injury to the brain results not only from ROS overproduction but also from inflammatory reactions in microglia and astrocytes. As a consequence of increased TNF-*α* secretion, the transcription factor NF-*κ*B (nuclear factor kappa-light-chain-enhancer of activated B cells) is overexpressed, which stimulates the synthesis of proinflammatory interleukins (IL-1*β*, IL-6), inducible nitric oxide synthase (iNOS), and adhesion molecules (VCAM, ICAM). This promotes adhesion and diapedesis of immunocompetent cells across the vascular endothelium. Increased NO bioavailability also stimulates the formation of peroxynitrite, which is one of the most potent oxidizing and nitrating agents. In the final stage, oxidative DNA damage and neuronal death occur, resulting in impairment of the cerebral cortex and other brain structures responsible for cognitive functions [75, 76, 117–122]. It was shown that ALA prevents TNF-*α*-induced activation of NF-*κ*B. Indeed, in our study, ALA decreases level of proinflammatory TNF-*α* but also increases the synthesis of anti-inflammatory IL-10 in the hypothalamus of insulin-resistant rats. This may be due to *Nrf2* gene activation [123], which inhibits *NF-κB* and thus the expression of many proinflammatory genes. ALA also reduces hypothalamic nitrosative damage (↓3-NT) and prevents neuronal apoptosis (↓casp-3), confirming its multidirectional effects within the brain. These observations are confirmed by reduced expression of proinflammatory and proapoptotic genes in RT-PCR (↓*NF-κB*, ↓*TNF-α*, and ↓*casp-3*) (Figure 11). It should not be forgotten that ALA is one of the coenzymes in the multienzyme dehydrogenase complexes, including pyruvate dehydrogenase and *α*-ketoglutarate dehydrogenase [124]. Although our study does not explain it, ALA may improve brain mitochondrial activity.

The protective effects of ALA are not limited to the brain. We showed that ALA normalizes body weight, BMI, glycemia, insulinemia, and HOMA-IR index of insulin-resistant rats. ALA also reduces the risk of cardiometabolic complications shown as a decrease in the Lee index. This confirms previous observations regarding the effects of ALA on glucose homeostasis and obesity. ALA reduces low-density lipoproteins (LDL), total cholesterol, and triglycerides, which is due to inhibition of free fatty acid accumulation and increased insulin sensitivity in diabetic patients [50–52]. Interestingly, we have shown no correlation between brain and serum/plasma oxidative stress biomarkers, indicating the different nature of redox imbalance at the central and systemic levels. Although further research is needed, ALA could find application in patients with obesity, insulin resistance, and type 2 diabetes. ALA supplementation has virtually no side effects. The main adverse reactions include skin allergy and, for diabetics, hypoglycemia at high doses of drug. The approximate lethal dose (LD_50_) is 400-500 mg/kg BW (regardless of route of administration), and long-term oral supplementation causes only weight loss [125, 126].

Unfortunately, our study also has some limitations. Due to the very small mass of the brain tissue, we could only determine selected redox, inflammatory, and apoptosis biomarkers. We also did not conduct cognitive studies in rats. Further studies are needed to evaluate the molecular mechanisms of ALA action within the insulin-resistant brain. Clinical studies on the effects of ALA on the cerebral complications of insulin resistance are also necessary.

## 5. Conclusions

1. ALA reduces body weight, normalizes glucose and insulin levels, and restores systemic insulin sensitivity in HFD-fed rats
2. ALA supplementation enhances enzymatic and nonenzymatic antioxidant systems and reduces oxidative damage to proteins, lipids, and DNA, mainly in the hypothalamus of insulin-resistant rats. The protective effects of ALA result from hypothalamic activation of the transcription factor Nrf2 and inhibition of NF-*κ*B
3. ALA improves systemic redox homeostasis; however, no relationship was found between local and central oxidative stress
4. ALA diminishes carbonyl/glycative stress, protein nitrosative damage, inflammation, and apoptosis in the hypothalamus of insulin-resistant rats but generally does not affect the cerebral cortex
5. Further studies are needed to determine the molecular mechanism of ALA action within the brain

## Data Availability

The datasets generated for this study are available on reasonable request to the corresponding author.

## Supplementary Materials

Supplementary Materials Figure S1: effect of ALA supplementation on body weight, BMI, Lee index, fasting plasma glucose, insulin, and HOMA-IR. Values are means ± SD, *n* = 10. Differences statistically significant at ^∗^*p* < 0.05 and ^∗∗∗∗^*p* < 0.0001. BMI: body mass index; CD ALA-: control animals not supplemented with ALA; CD ALA+: control animals supplemented with ALA; HFD ALA-: high-fat diet-fed animals not supplemented with ALA; HFD ALA+: high-fat diet-fed animals supplemented with ALA; HOMA-IR: homeostatic model assessment of *β*-cell function and insulin resistance. Figure S2: effect of ALA supplementation on serum/plasma level of enzymatic (CAT, GPx, GR, and SOD) and nonenzymatic (total glutathione, GSH, GSSG, and redox potential) antioxidants. Values are means ± SD, *n* = 10. Differences statistically significant at ^∗^*p* < 0.05, ^∗∗^*p* < 0.005, ^∗∗∗^*p* < 0.0005, and ^∗∗∗∗^*p* < 0.0001. CAT: catalase; CD ALA-: control animals not supplemented with ALA; CD ALA+: control animals supplemented with ALA; GPx: glutathione peroxidase; GR: glutathione reductase; GSH: reduced glutathione; GSSG: oxidized glutathione; HFD ALA-: high-fat diet-fed animals not supplemented with ALA; HFD ALA+: high-fat diet-fed animals supplemented with ALA; SOD: superoxide dismutase-1. Figure S3: effect of ALA supplementation on the plasma level of oxidative damage markers (PC, AOPP, AGE, 4-HNE, 8-izoprostanes, and 8-OHdG). Values are means ± SD, *n* = 10. Differences statistically significant at ^∗^*p* < 0.05, ^∗∗^*p* < 0.005, ^∗∗∗^*p* < 0.0005, and ^∗∗∗∗^*p* < 0.0001. AGE: advanced glycation end products; AOPP: advanced oxidation protein products; CD ALA-: control animals not supplemented with ALA; CD ALA+: control animals supplemented with ALA; HFD ALA-: high-fat diet-fed animals not supplemented with ALA; HFD ALA+: high-fat diet-fed animals supplemented with ALA; 4-HNE: 4-hydroxynonneal protein adducts; 8-OHdG: 8-hydroxy-2′-deoxyguanosine; PC: protein carbonyl groups.
