# Supplementary material for: α-Lipoic Acid Strengthens the Antioxidant Barrier and Reduces Oxidative, Nitrosative, and Glycative Damage, as well as Inhibits Inflammation and Apoptosis in the Hypothalamus but Not in the Cerebral Cortex of Insulin-Resistant Rats
Source: Oxid Med Cell Longev. 2022 Mar 29;2022:7450514. doi: 10.1155/2022/7450514 (PMC8983239; doi:10.1155/2022/7450514)
Supplement: Supplementary Materials — Figure S1: effect of ALA supplementation on body weight, BMI, Lee index, fasting plasma glucose, insulin, and HOMA-IR. Values are means ± SD, n = 10. Differences statistically significant at ∗p < 0.05 and ∗∗∗∗p < 0.0001. BMI: body mass index; CD ALA-: control animals not supplemented with ALA; CD ALA+: control animals supplemented with ALA; HFD ALA-: high-fat diet-fed animals not supplemented with ALA; HFD ALA+: high-fat diet-fed animals supplemented with ALA; HOMA-IR: homeostatic model assessment of β-cell function and insulin resistance. Figure S2: effect of ALA supplementation on serum/plasma level of enzymatic (CAT, GPx, GR, and SOD) and nonenzymatic (total glutathione, GSH, GSSG, and redox potential) antioxidants. Values are means ± SD, n = 10. Differences statistically significant at ∗p < 0.05, ∗∗p < 0.005, ∗∗∗p < 0.0005, and ∗∗∗∗p < 0.0001. CAT: catalase; CD ALA-: control animals not supplemented with ALA; CD ALA+: control animals supplemented with ALA; GPx: glutathione peroxidase; GR: glutathione reductase; GSH: reduced glutathione; GSSG: oxidized glutathione; HFD ALA-: high-fat diet-fed animals not supplemented with ALA; HFD ALA+: high-fat diet-fed animals supplemented with ALA; SOD: superoxide dismutase-1. Figure S3: effect of ALA supplementation on the plasma level of oxidative damage markers (PC, AOPP, AGE, 4-HNE, 8-izoprostanes, and 8-OHdG). Values are means ± SD, n = 10. Differences statistically significant at ∗p < 0.05, ∗∗p < 0.005, ∗∗∗p < 0.0005, and ∗∗∗∗p < 0.0001. AGE: advanced glycation end products; AOPP: advanced oxidation protein products; CD ALA-: control animals not supplemented with ALA; CD ALA+: control animals supplemented with ALA; HFD ALA-: high-fat diet-fed animals not supplemented with ALA; HFD ALA+: high-fat diet-fed animals supplemented with ALA; 4-HNE: 4-hydroxynonneal protein adducts; 8-OHdG: 8-hydroxy-2′-deoxyguanosine; PC: protein carbonyl groups. [file 7450514.f1.docx]

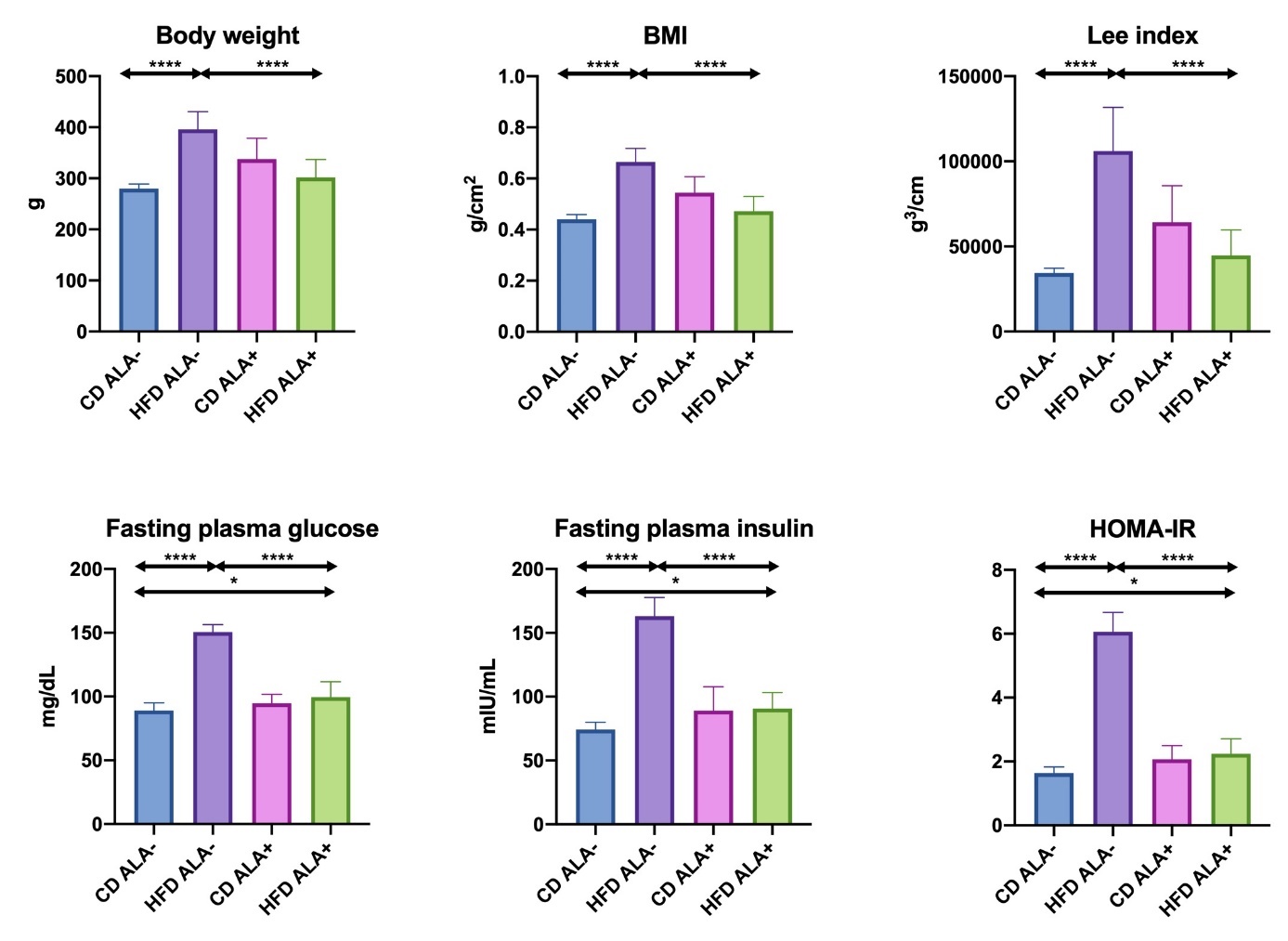


**Figure S1.** Effect of ALA supplementation on body weight, BMI, Lee index, fasting plasma glucose, insulin and HOMA-IR. Values are means ± SD, n = 10. Differences statistically significant at: * p < 0.05, **** p < 0.0001. BMI, body mass index; CD ALA- , control animals not supplemented with ALA; CD ALA+, control animals supplemented with ALA; HFD ALA-, high fat diet-fed animals not supplemented with ALA; HFD ALA+, high fat diet-fed animals supplemented with ALA; HOMA-IR, homeostatic model assessment of β-cell function and insulin resistance

**
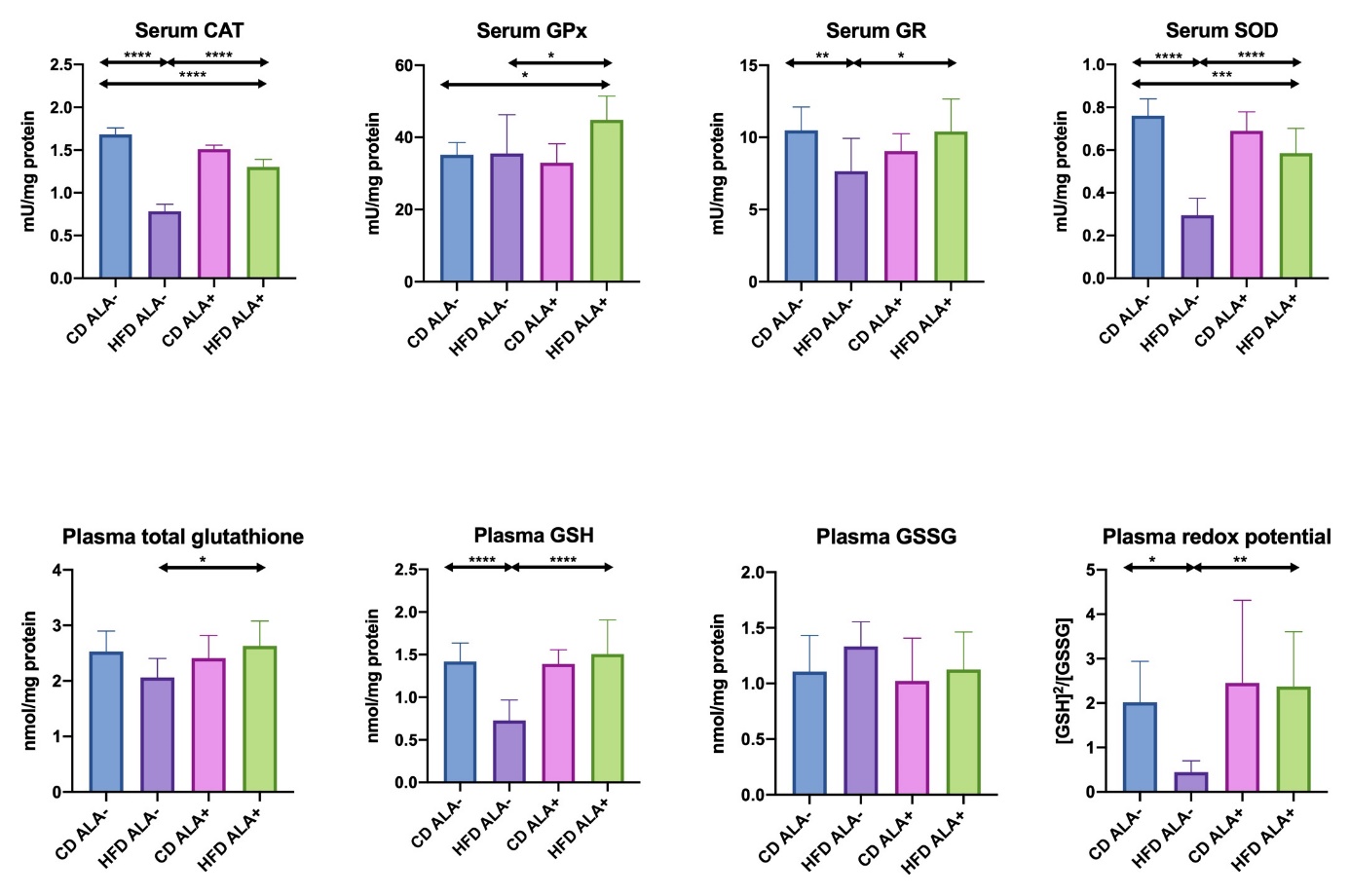
**

**Figure S2.** Effect of ALA supplementation on serum/plasma level of enzymatic (CAT, GPx, GR and SOD) and non-enzymatic (total glutathione, GSH, GSSG and redox potential) antioxidants. Values are means ± SD, n = 10. Differences statistically significant at: * p < 0.05, ** p < 0.005, *** p < 0.0005, **** p < 0.0001. CAT, catalase; CD ALA- , control animals not supplemented with ALA; CD ALA+, control animals supplemented with ALA; GPx, glutathione peroxidase; GR, glutathione reductase; GSH, reduced glutathione; GSSG, oxidized glutathione; HFD ALA-, high fat diet-fed animals not supplemented with ALA; HFD ALA+, high fat diet-fed animals supplemented with ALA; SOD, superoxide dismutase-1.


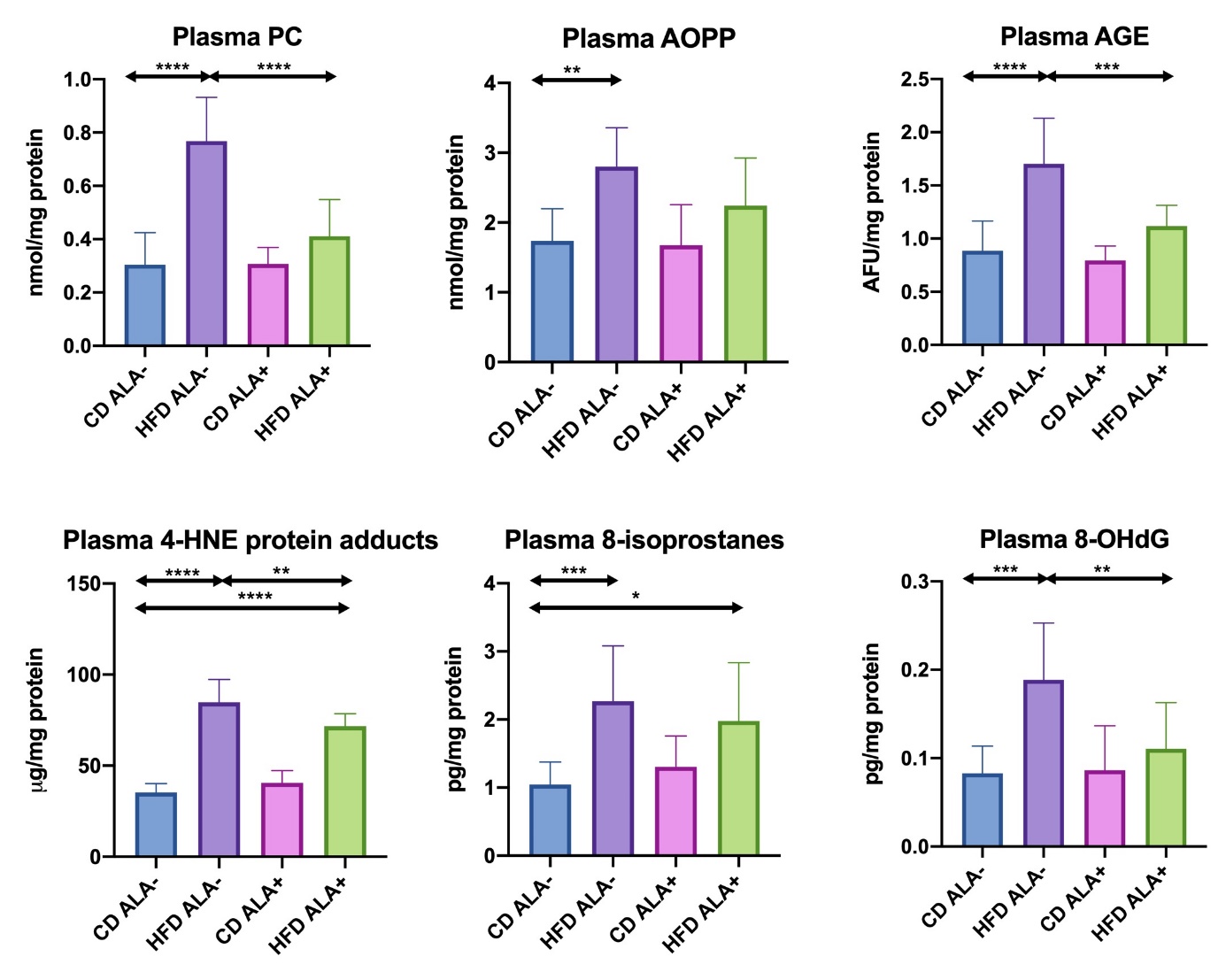


**Figure S3.** Effect of ALA supplementation on plasma level of oxidative damage markers (PC, AOPP, AGE, 4-HNE, 8-izoprostanes and 8-OHdG). Values are means ± SD, n = 10. Differences statistically significant at: * p < 0.05, ** p < 0.005, *** p < 0.0005, **** p < 0.0001. AGE, advanced glycation end products; AOPP, advanced oxidation protein products; CD ALA- , control animals not supplemented with ALA; CD ALA+, control animals supplemented with ALA; HFD ALA-, high fat diet-fed animals not supplemented with ALA; HFD ALA+, high fat diet-fed animals supplemented with ALA; 4-HNE, 4-hydroxynonneal protein adducts; 8-OHdG, 8-hydroxy-2′-deoxyguanosine; PC, protein carbonyl groups.
